# Improving the Anti-washout Property of Acrylate Grouting Material by Bentonite: Its Characterization, Improving Mechanism, and Practical Application

**DOI:** 10.3390/polym15193865

**Published:** 2023-09-23

**Authors:** Zuochun Li, Feng Huang, Yuyou Yang, Yifan Xiong, Fei Su, Yajian Wang, Xiao Tian

**Affiliations:** 1 School of Engineering and Technology, China University of Geosciences (Beijing), Beijing 100083, China; lizuochun@cugb.edu.cn (Z.L.); yangyuyou@cugb.edu.cn (Y.Y.); 2102190059@cugb.edu.cn (Y.X.); wangyj@cugb.edu.cn (Y.W.); tianx@cugb.edu.cn (X.T.); 2 China Institute of Nuclear Industry Strategy, Beijing 100045, China; feisuchina@126.com

**Keywords:** polymer slurry, bentonite, dynamic water grouting, acrylate, treatment of groundwater seepage

## Abstract

Acrylate is a popular polymer grouting material that has been widely used to control groundwater seepage. However, the vulnerability of acrylate slurry to dynamic water washout restricts its application in groundwater environments characterized by high flow velocity and water pressures. In this paper, lithium bentonite (Li-B) was used to modify the traditional magnesium acrylate (AC) grouting material. The influence of Li-B to AC ratios on the modified materials’ washout resistance was explored, and the modification mechanism was analyzed using X-ray diffraction (XRD), infrared spectroscopy (IR), and scanning electron microscopy (SEM). Finally, the anti-washout ability of the modified slurry was verified through engineering applications. Results revealed that LiB-AC grout had adjustable setting times (10.5 to 395.6 s), minimal bleeding (0.1%), higher viscosity (65 mPa·s) and expansibility (350%), stronger anti-water dispersibility (24 times that of pure AC slurry), higher mechanical strength (compressive strength is 0.386 MPa, tensile strength is 0.088 MPa), and better impermeability (2.23 × 10^−8^ m/s). The lithium bentonite was beneficial to the setting time, bleeding, viscosity, slurry retention rate, impermeability, and mechanical strength of the acrylate grout. However, it diminished the expansibility of the acrylate grout. At the optimal acrylate content (20%), the mechanical strength and impermeability of the LiB-AC grout were the highest. The better performance of LiB-AC grout was attributed to the formation of a more stable and dense interlaced spatial network structure after the modification by Li-B. The LiB-AC grout was used in the dynamic water grouting project of a metro shield tunnel segment and achieved better anti-washout performance than cement-water glass and pure AC slurry.

## 1. Introduction

Structural aging and rising groundwater levels may subject underground infrastructure to groundwater leakage and sudden surges [1,2,3]. Grouting is an effective method for dealing with these accidents, and selecting appropriate grouting materials is of great significance [4,5,6]. Acrylate slurries feature low viscosity, adjustable setting times, and a low permeability coefficient. They also have strong adaptability in both large-pore strata and small fissures, making them widely applicable in controlling groundwater leakage [7,8,9]. However, the acrylate grouting materials’ weak resistance against dynamic water washout restricts their utility in tunnels in groundwater environments characterized by high flow velocity and water pressure [10,11].

There are three main approaches to enhancing a slurry’s resistance to dynamic water washout. The first approach is to accelerate the slurry’s reaction rate to facilitate the formation of stones or gels, thereby bolstering its resistance to dynamic water washout [12,13,14]. However, an excessively fast reaction rate may hinder the diffusion of the slurry within the formation and lead to blockages in grout pipes, leading to failure to achieve expected results. The second method is to select a suitable grouting process to improve the grouting effectiveness in a dynamic water environment, such as the grouting pressure, the size, depth, number, and layout shape of the grouting holes, etc. [15,16]. Unfortunately, this method does not fundamentally address the weak dispersion resistance of the material itself, resulting in the method no longer being applicable in high flow velocity and water pressure environments [17,18]. At the same time, due to the complexity of the procedure, the method needs to be individually designed according to the specific project, which leads to its poor universality [19]. The last method is to use anti-dispersion agents to improve the anti-dispersion of a grouting material. These agents primarily consist of water-soluble polymers that exploit the thickening effect to augment the anti-dispersion performance [20,21]. Scholars have found that many anti-dispersion materials can improve the anti-washout ability of various slurries, such as Veolan gum, cellulose, polypropylene fiber, xanthan gum, etc. [22,23,24].

Up until now, the above anti-dispersion materials were aimed primarily at cement-based grout. There is limited research on suitable anti-dispersion materials for Acrylate slurries.

Bentonite has excellent thickening, anti-dispersion, high adsorption, and environmental performance, making it one of the most promising modified materials [25,26,27,28]. Bentonite has been widely used to improve the viscosity, bleeding characteristics, and impermeability of cement-based slurry, as well as the absorbency and gel strength of polymer absorbent resins [29,30,31,32,33]. However, there is relatively little research on the effect of bentonite on the performance of Acrylate slurry, and the modification mechanism of bentonite on Acrylate slurry is not clear, which motivates the authors to conduct this research.

In this study, bentonite was used to improve the anti-dispersion performance of acrylate slurry. Firstly, lithium bentonite was used to modify the acrylate slurry. Many laboratory experiments were conducted to study the lithium bentonite-acrylate grout’s properties with varying ratios (wt%) of lithium bentonite to acrylate. The properties were the slurry’s apparent viscosity, bleeding rate, setting time, expansibility, resistance to dynamic water washout, impermeability, mechanical strength, and microstructure. Afterwards, the modification mechanism was investigated and discussed using X-ray diffraction (XRD), infrared spectroscopy (IR), and scanning electron microscopy (SEM). Finally, the lithium bentonite-acrylate grout’s effectiveness in resisting dynamic water washout was verified through engineering applications.

## 2. Materials and Methods

### 2.1. Materials

The bentonite used in the experiment was Li-bentonite (Li-B), which was bought from the Lingshou County Langtai Mineral Products Processing Plant (Shijiazhuang, China). Its chemical composites are shown in Table 1.

The main agents, cross-linking agent, initiator, and emulsifier used in the mixture were magnesium acrylate (AC), N, N’-methylenebis (MBA), ammonium persulfate (APS), and sodium dodecyl benzene sulfonate (SDBS), respectively, which were bought from Shanghai Macklin Biochemical Co., Ltd. (Shanghai, China). Moreover, the accelerator used in the mixture was triethanolamine (TEA), which was bought from Shandong Yousuo Chemical Technology Co., Ltd. (Linyi, China). The purity specifications of the above chemical reagents are all Analytical Reagent (AR).

### 2.2. Mixing and Testing Procedures

**Figure 1:**
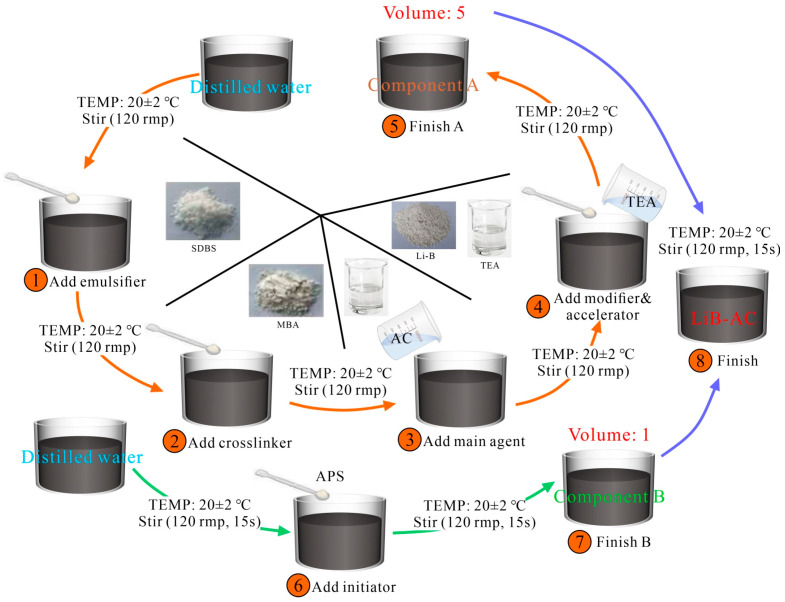
Preparation process of LiB-AC grout.

Component A is a mixture of AC solution, MBA, TEA, SDBS, Li-B, and distilled water in a certain ratio. Component B is a solution that consists of APS and distilled water. The volume ratio of components A and B is 5:1. The preparation process of LiB-AC grout is shown in Figure 1. When grouting in water-rich strata, the AC content usually needs to be greater than 15%. Meanwhile, after the bentonite content exceeds 40%, the slurry will be difficult to stir. Therefore, the AC dosage was designed as 15, 20, and 25 wt%, and the Li-B dosage was designed as 5, 10, 15, 20, 25, and 30 wt%.

Component A: distilled water, AC, MBA, SDBS, Li-B, and TEA were added to the beaker in turn and mixed by an electric mixer at a rotation speed of 120 rpm. Continue stirring until mixed with component B to prevent Li-B settling.

Component B: APS was introduced into the distilled water and mixed by an electric mixer at a rotation speed of 120 rpm. After stirring for 15 s, components A and B were mixed at a volume ratio of 5:1 for 15 s at a rotation speed of 120 rpm.

The apparent viscosity, bleeding rate, setting time, expansion characteristic, retention rate of slurry, impermeability, compressive strength, tensile strength, and microstructure analysis were tested as described below.

#### 2.2.1. Apparent Viscosity

The apparent viscosity characteristic of LiB-AC grout was tested using ZNN-D6. The spindle was immersed in the paste and rotated at a speed of 600 rpm, and the 1/2 value of the reading was taken as the apparent viscosity of the slurry.

#### 2.2.2. Bleeding Rate

**Figure 2:**
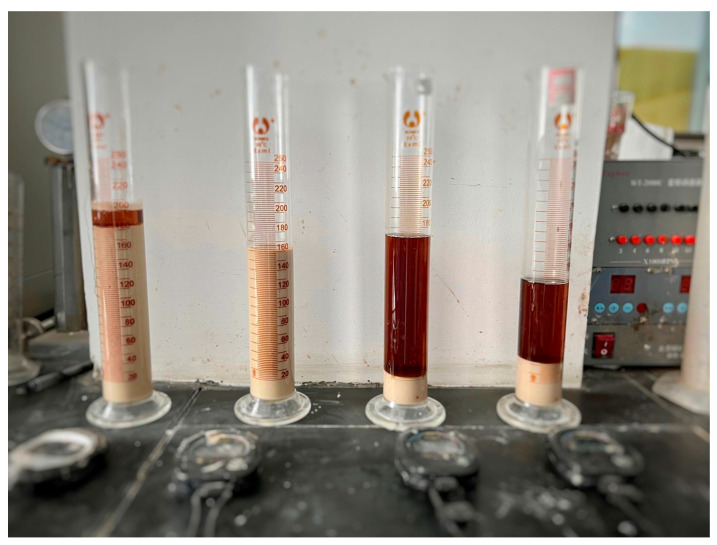
Bleeding test of LiB-AC grout.

The bleeding test was adopted to characterize the stability of LiB-AC grout. It was conducted with a graduated cylinder (250 mL), as shown in Figure 2. LiB-AC grout was injected into a graduated cylinder. The scale between the clear upper water and the lower sediment was recorded at 10, 30, 60, and 120 min. The bleeding rate is represented by the ratio of the volume of water bleeding from the grout to the total volume of the LiB-AC grout.

#### 2.2.3. Setting Time

**Figure 3:**
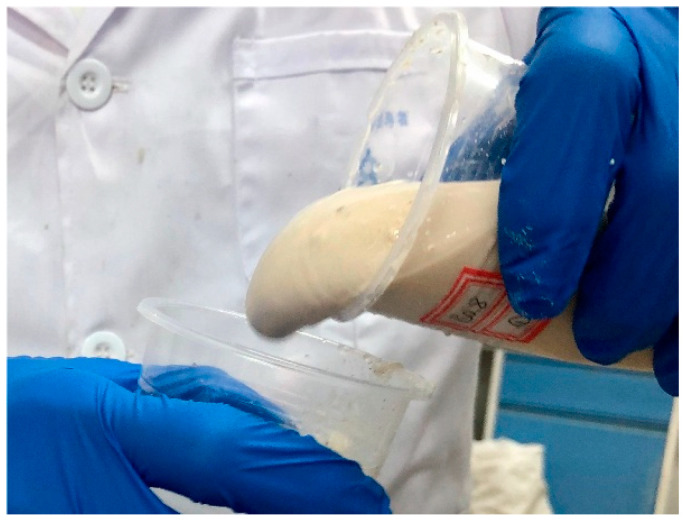
Setting time test of LiB-AC grout.

The ambient temperature for this test should be kept at 20 ± 2 °C. As the setting time of LiB-AC grout is too short, the inverted cup method was adopted to test the initial setting time, as shown in Figure 3. A certain amount of components A and B were placed in two plastic cups, respectively. The two plastic cups were repeated alternately and poured until the slurry completely lost fluidity.

#### 2.2.4. Retention Rate of Slurry

**Figure 4:**
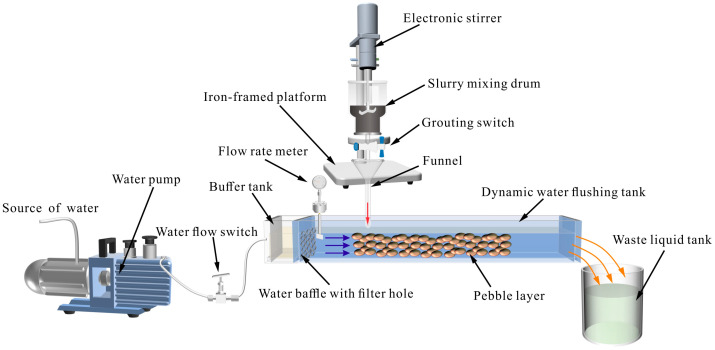
The testing device for slurry retention rate.

The testing device for the slurry retention rate is shown in Figure 4. The water pump was used to provide the dynamic water at different flow velocities, and the water baffles with filter holes were designed to keep flow velocity uniform. LiB-AC grout was stirred quickly for 15 s, and then the grouting switch was opened. The test was conducted for 1 min. After the test, the water in the tank was drained. The mass of the slurry retained in the pebble stratum was obtained. The test was carried out using a single-factor controlled variable method with different flow velocities and slurry ratios, and each group was repeated three times. The retention rate is represented by the ratio of the mass of the slurry retained in the pebble stratum to the total mass of the slurry.

#### 2.2.5. Expansion Characteristic

**Figure 5:**
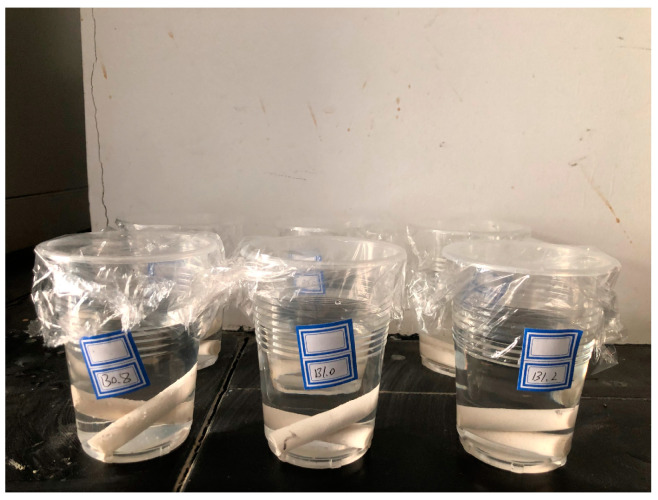
Expansion characteristics test of LiB-AC grout.

The dimensions of the expansion-characteristic specimen were Φ 10 × 50 mm (Figure 5). The specimens were de-molded after one hour of curing, and they were stored in distilled water at a constant temperature of 20 ± 2 °C. The volume of the specimen was tested by the drainage method. Three specimens were tested to obtain an average volume change. The expansion rate of LiB-AC grout curing for 1, 3, and 7 d The expansion rate is represented by the ratio of the volume change of the specimen at different curing times and the initial volume of the specimen.

#### 2.2.6. Impermeability

**Figure 6:**
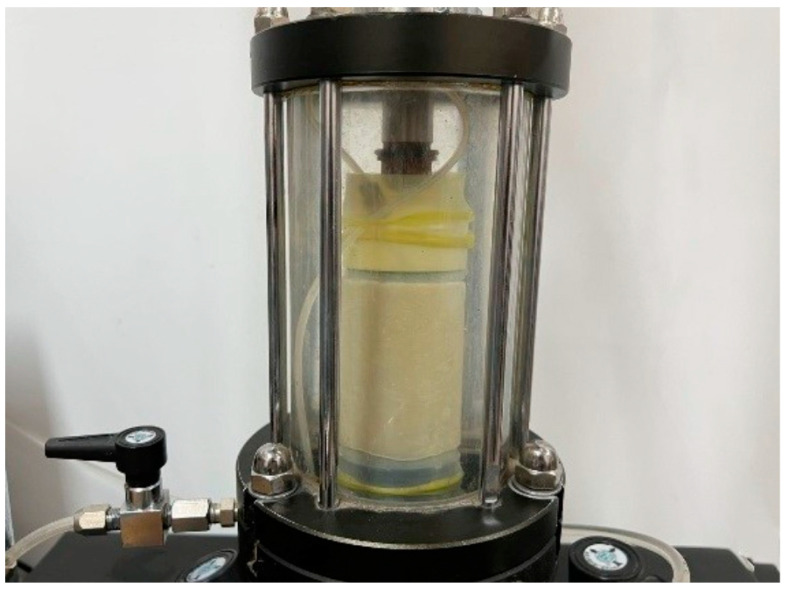
Impermeability test of LiB-AC grout.

The impermeability of LiB-AC grout was tested using a stress-strain-controlled triaxial shear permeability tester (SLB-1), as shown in Figure 6. The dimensions of the impermeability specimen were Φ 39.1 × 80 mm, and they were de-molded after 24 h of curing. Three specimens were tested to obtain an average permeability coefficient.

#### 2.2.7. Compressive Strength Test

**Figure 7:**
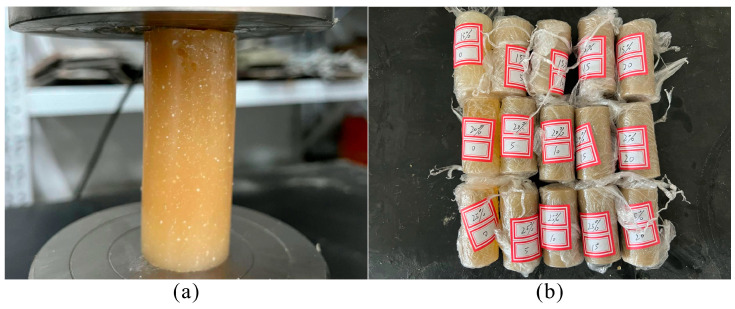
Compressive strength test of LiB-AC grout: (**a**) the process of compressive strength test; (**b**) specimens for compressive strength test.

The compressive strength of LiB-AC grout was tested on 40-mm cylinders according to ASTM C942 [34]. The grouts were cast into Φ 40 × 100 mm molds (Figure 7). The specimens were de-molded after 24 h and then cured in moist conditions with a constant temperature of 20 ± 2 °C and 95% relative humidity for 1, 3, and 7 d. Each group of specimens was tested six times. Removed the maximum and minimum values, and the average compressive strength was obtained.

#### 2.2.8. Tensile Strength

**Figure 8:**
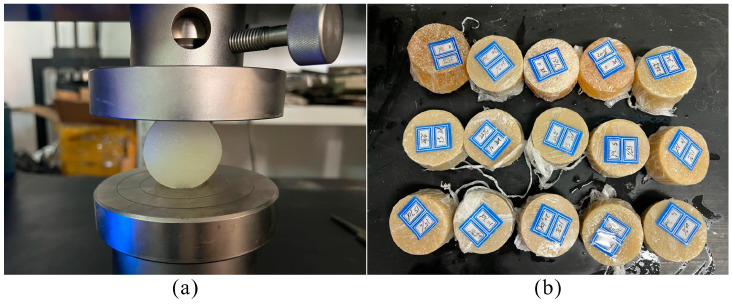
Tensile strength test of LiB-AC grout: (**a**) the process of tensile strength test; (**b**) specimens for tensile strength test.

The Brazilian splitting method was adopted to test the tensile strength. The tensile strength of LiB-AC grout was tested on 50-mm cylinders according to ASTM C496 [35]. The grouts were cast into Φ 50 × 25 mm molds (Figure 8). The method for demolding, curing, and average value calculation of the specimen is the same as the compressive strength test.

#### 2.2.9. Microstructure Analysis

The chemical bonds of the specimens were determined by AXS D8-Focus from Bruker, Rheinstetten, Germany, and FTIR-8000S from Seiko, Tokyo, Japan. The microstructures of LiB-AC grout were investigated by Scanning Electron Microscopy (SEM, COXEM EM-30Plus, Daejeon, Republic of Korea). All specimens of microstructure analyzed were crushed into pieces using the stone body after the mechanical test, then stored in alcohol and dried at 60 °C for 24 h before microstructure analysis.

## 3. Results and Discussion

### 3.1. Workability

#### 3.1.1. Apparent Viscosity

**Figure 9:**
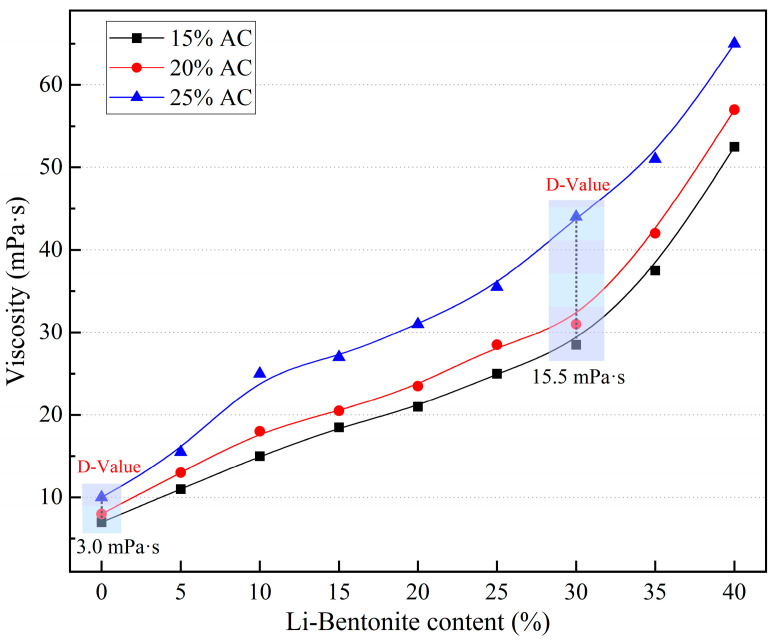
The apparent viscosity of LiB-AC grout.

The viscosity of the slurry is a crucial parameter in grouting engineering. Apparent viscosity directly affects the fluidity and pumpability of slurry, and it can be used to guide grouting pressure design and slurry pumping distance determination. The apparent viscosity of LiB-AC grout with different admixtures of AC and Li-B is shown in Figure 9.

Figure 9 shows the apparent viscosity evolution of LiB-AC grout with different contents of Li-B (0–40%) and AC (15–25%). It can be seen that the apparent viscosity of LiB-AC grout increased with the content of Li-B, and the viscosity value of 25% AC is high. In comparison, the viscosities of 15% AC and 20% AC are relatively low. It is because with the increase in AC content, the proportion of water in the slurry is relatively reduced, and the content of AC macromolecules in the slurry increases, resulting in a gradual increase in the viscosity of the slurry. The viscosity of the slurry increases slowly in the 0–30% Li-B content interval at 15% and 20% AC content and accelerates at 30% to 40%. Compared with the 15% AC and 20% AC groups, the viscosity of the slurry in the 25% AC group increased significantly faster, which suggests that as the AC content increased, its impact became more dominant.

Furthermore, the thickening effect of Li-B on the LiB-AC grout was more significant than the effect of AC. The effect of AC content on the slurry viscosity was most significant at 30% Li-B, with an increase of 54.4% in the slurry viscosity in the 25% AC group relative to the 15% AC group. However, by varying the Li-B content in the slurry, the maximum increase in slurry viscosity was 650%, 612.5%, and 550% for the 15%, 20%, and 25% AC groups, respectively. This is because the Li-B particles and the AC polymer adsorb each other interleaved, thus forming an interleaved spatial mesh structure, significantly increasing the slurry viscosity. The above results show that in the interval of 0–40%, the higher the content of Li-B, the higher the viscosity of the slurry. Nonetheless, high viscosity indicators indicate poor fluidity in the slurry. Therefore, the viscosity of the slurry should be adjusted according to the speed and flow rate of the water flow in the actual dynamic water grouting project.

#### 3.1.2. Bleeding

**Figure 10:**
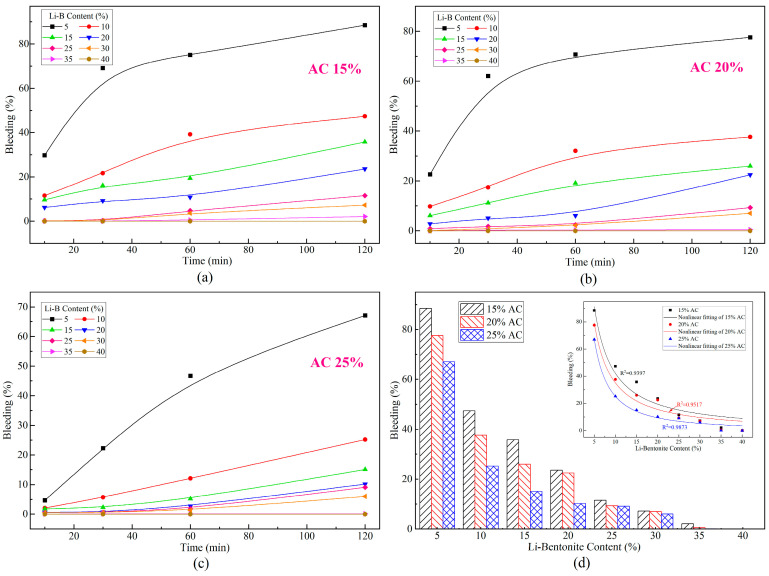
The bleeding rate of LiB-AC grout: (**a**) 15% AC content in grout; (**b**) 20% AC content in grout; (**c**) 25% AC content in grout; (**d**) bleeding rate variation curve of grout with different AC and bentonite content.

The bleeding of slurry influences the filling and flow behavior of slurry injected into soil and structural cracks. The bleeding of LiB-AC grout with different admixtures of Li-B and AC is shown in Figure 10.

It can be observed from Figure 10 that the bleeding of the slurry gradually increases with time. The admixture of Li-B has a positive role in reducing the bleeding rate, which is consistent with the previous studies [36,37]. As the content of bentonite increases, the inhibitory effect of bentonite on bleeding evolution gradually weakens. The growth rate of bleeding is relatively large when the bentonite content is less than 20%, and the growth rate becomes smaller when the bentonite content is higher than 25%. This phenomenon is most apparent in the bentonite content of the 5% group. It is owing to the positive charge and hydration radius of small cations being relatively greater as the bentonite content is low that the negative electric repulsion between the montmorillonite particles is relatively weak, and the bentonite particles are prone to form larger polymers with acrylate. As a result, the slurry appears to have poor suspension and a high bleeding rate. However, the negatively charged montmorillonite particles gradually increased as Li-B content increased, which reduced the van der Waals force between the lamellas. Meanwhile, due to the lamellae becoming more easily dispersed and flaked, the repulsive force between the lamellae of the AC polymer suspension is enhanced when the lamellae are bonded to the AC polymer. Consequently, the bleeding of the slurry is significantly reduced.

Furthermore, it can be concluded from Figure 10 that the sensitivity of the slurry bleeding to bentonite content decreases after the bentonite content is greater than 25%. It is suggested that Li-B had an optimal content value, and its effect on slurry bleeding was no longer apparent after exceeding the value. Besides, adding a large amount of bentonite would affect its fluidity. Therefore, in practical applications, an appropriate amount of bentonite is selected based on the actual situation on site. It not only improves the stability of the slurry but also enhances its pumping ability.

Similarly, as the AC content increases, the bleeding value of the slurry decreases, as shown in Figure 10d. Moreover, as the bentonite content of the slurry increases, this effect will gradually diminish. It indicates that bentonite content plays a dominant role in influencing the bleeding of the slurry. This is because lamellae mainly influence the suspension of the polymer in the slurry. Although the AC skeleton can also play a certain role in suspension, due to its intermolecular gravity, the suspension effect is not as significant as Li-B. Besides, from the nonlinear fitting curves in Figure 10d, it can be concluded that as the bentonite content in the slurry exceeds 20%, the effect of AC on the bleeding value can be almost ignored. Hence, the content of AC can be appropriately reduced when it satisfies the viscosity of LiB-AC grout.

#### 3.1.3. Setting Time

**Figure 11:**
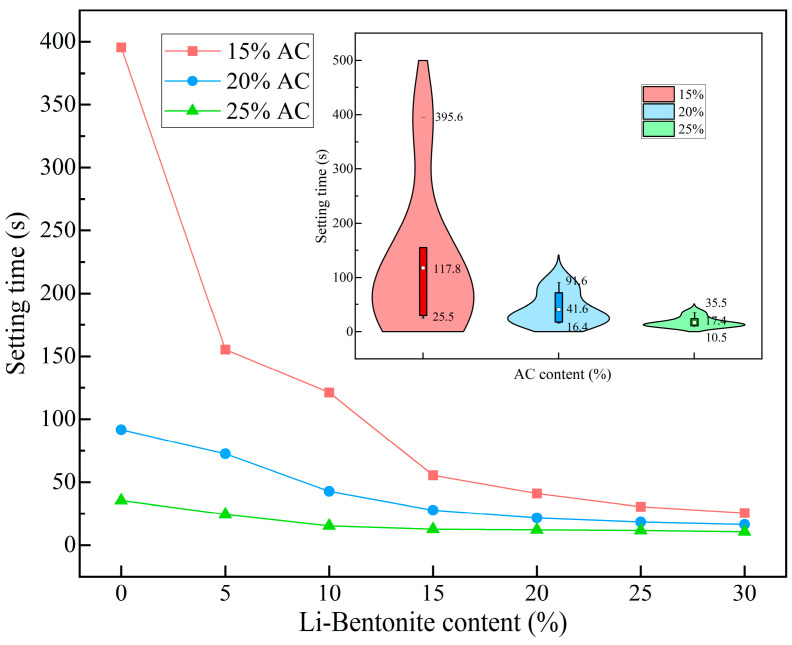
The setting time of LiB-AC grout.

The setting time of the grout material determines its pump ability and engineering utility. In dynamic water sealing projects, grout should be characterized by an adjustable setting time (a few seconds to a few minutes) to achieve the sealing effect. As shown in Figure 11, within the designed experimental scheme range, the setting time of LiB-AC grout was 10.5 s to 395.6 s.

The change in setting time of LiB-AC was mainly related to the chemical reactions, such as AC polymerization and the grafting reaction of AC and Li-B, as well as physical changes, such as moisture gain and the packing effect of Li-B. When the content of bentonite is constant, the setting time of the slurry becomes shorter with the increase in AC content (as shown in Figure 11). When the Li-B content was small, the difference in setting time between the groups with different AC contents was significant. As the content of bentonite increases, this difference sharply decreases. This result may be related to the fact that when the AC content increases, the double bond active group in the reaction system increases, and the growth rate of the initiation chain increases, which accelerates the viscosity growth of the slurry (the number of macromolecular chains increases and the flow is blocked), which leads to the shortening of setting time.

Similarly, when AC content is constant, the setting time of the slurry decreases with the increase in Li-B content. When AC content is low (15%), the setting time of the slurry changes significantly with the increase in bentonite. With the increase in AC content, the influence of bentonite on the slurry setting time gradually weakens. The increase in bentonite content causes a relative decrease in the water content; therefore, the number of molecules in the AC unit volume increases, which makes it easier for the double bonds to polymerize. The Li-B and AC are also intercalated and stacked in lamellae, making the molecules more compact and easier to polymerize. Therefore, the setting time was reduced. Moreover, from the violin chart in Figure 11, it can be seen that when AC content is low, with the change in bentonite content, the setting time changes considerably. This phenomenon gradually decreases with the increase in AC content, and the setting time tends to be stable.

**Figure 12:**
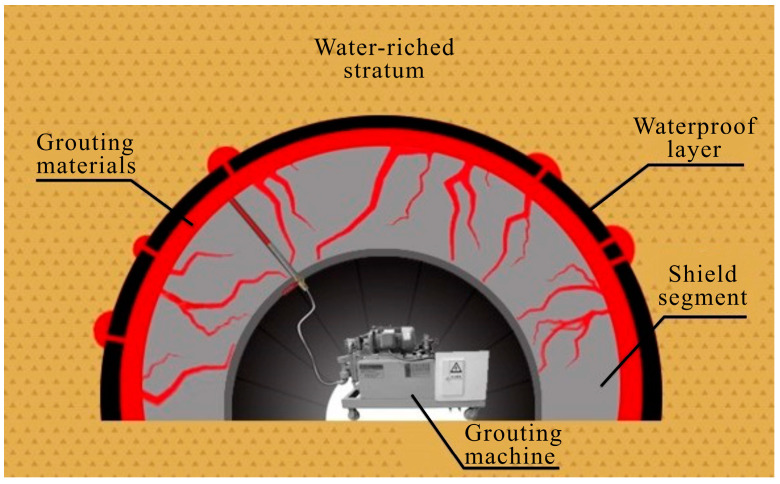
The backpack grouting technique.

In addition, suitable grouting technology should be selected to match the LiB-AC grouting material to guarantee the effect of dynamic water sealing. Preferably, a backpack grouting machine was adopted in the dynamic water grouting engineering (Figure 12). As the new grout has a short setting time, in order to achieve a fast and efficient dynamic water sealing effect, appropriate mixing methods must be selected to prevent pipe blockage accidents.

#### 3.1.4. Retention Rate of Slurry

**Figure 13:**
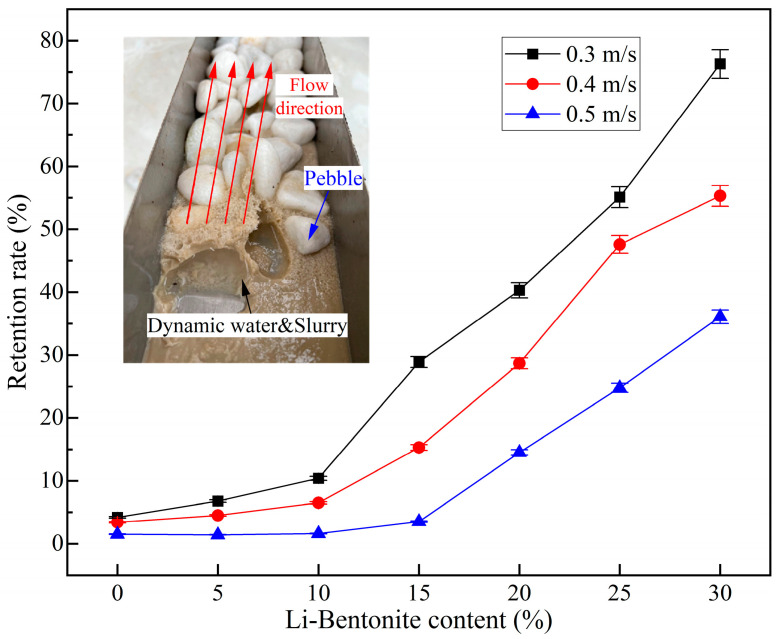
Retention rate of LiB-AC grout.

Grouting materials have good anti-dispersion performance, which is an essential guarantee for the grouting effect of dynamic water treatment works. The slurry retention rate of LiB-AC grout with different admixtures of Li-B and the flow velocity of dynamic water are shown in Figure 13.

From Figure 13, it can be concluded that Li-B played a positive role in the retention rate. When the Li-B content is 0%, the viscosity of the slurry is small, and the retention rate of the slurry is low under the circumstances of dynamic water. When the bentonite content in the slurry is less than 10%, the improvement of the slurry’s resistance to dynamic water washout by bentonite is not significant. With the increase in bentonite content in the slurry, the maximum retention rate can reach 76.3% when the Li-B content reaches 30%. Compared to low-content bentonite slurry, its resistance to dynamic water washout is greatly improved. It may be because Li-B and AC adhere to each other in the polymer formed by LiB-AC grout. The spatial network structure formed by their connections dramatically improves the stability and resistance to dynamic water washout. This phenomenon is consistent with viscosity, bleeding rate, and setting time.

Unsurprisingly, as the velocity of water flow increases, the retention rate of the slurry decreases. However, when the groundwater flow velocity is 0.5 m/s, the retention rate of the slurry is still 36.1%. Meanwhile, it can be observed from Figure 13 that LiB-AC grout can be rapidly condensed and tightly bonded with pebbles after flowing into the tank, effectively blocking the flow channel of the dynamic water.

### 3.2. Expansion Characteristic

**Figure 14:**
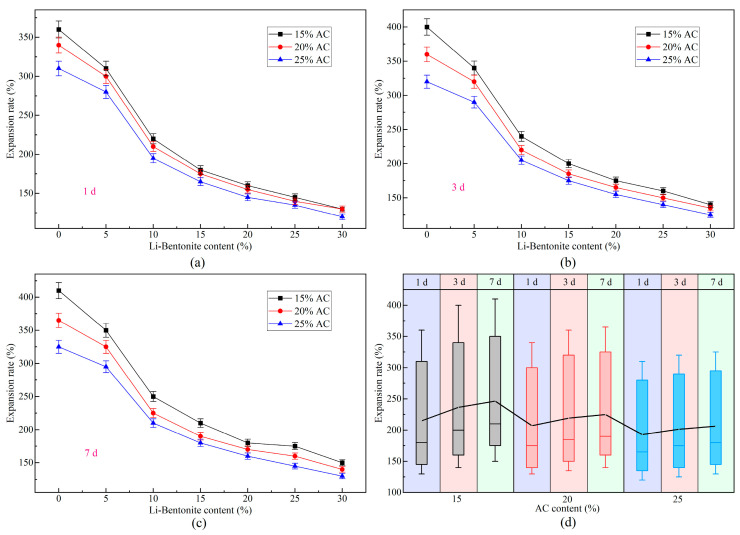
Expansion rate of LiB-AC grout. (**a**) curing time 1 day; (**b**) curing time 3 day; (**c**) curing time 7 day; (**d**) expansion rate of grout gel with different AC content under different curing time.

During the dynamic water grouting process, the expansion characteristic of the slurry’s gel is crucial for the effectiveness and durability of the engineering. If the expansion rate is minimal and even shrinkage occurs, there will be many gaps between the stratum and structure, leading to grouting failure. The expansion rate of LiB-AC grout with different admixtures of AC and Li-B is shown in Figure 14.

It can be observed from Figure 14a–c that LiB-AC grout has excellent expansion performance. The expansion rate of LiB-AC reaches a maximum of 410% and a minimum of 120%. This may be attributed to the variation in the spacing of the polymer lamellae. On the one hand, crystal lamella expansion occurs inside the slurry gel. It is because water is adsorbed in water molecular layers and linked in hydrogen bonds under the hydration of interlayer cations within the polymer. As interlayer water increases, many water molecules form a layer of water molecules that fills the interlayer, weakening the interlayer forces. As a result, the lamellar spacing increases, and crystal lamella expansion occurs. On the other hand, when the spacing increases to a certain extent, the difference in ion concentration between inside and outside the crystal lamella causes an osmotic pressure difference. This makes the water molecules enter the interlayer, and cations diffuse into the water, forming a double layer and generating repulsive forces. As a result, the lamellar spacing of the polymer increases, leading to osmotic expansion.

Furthermore, with the increase in bentonite content, the expansion rate of the gel presented an overall decreasing trend, as shown in Figure 14a–c. It may be due to the relative increase in hydrophilic ions in the polymer network structure as the content of bentonite increases. Therefore, the electrostatic repulsion between ions increases, and the water retention ability of the cross-linked network decreases. Meanwhile, when the content of bentonite is small, the water content in the polymer structure also decreases. It leads to osmotic expansion of the gel, and the expansion rate increases accordingly. In the same way, with the increase in AC content, the expansion rate also shows a downward trend. However, the effect of AC on the expansion rate of slurry is not as significant as that of bentonite. It is suggested that Li-B produces a more considerable electrostatic repulsive effect in the polymer lamellae than AC.

Moreover, from Figure 14d, it can be concluded that as the gel’s curing time grows, the slurry’s expansion rate increases. The expansion rate of the gel grows faster from 0 to 1 day and slows down from 1 to 3 and from 3 to 7 days. It is because, in the beginning, the gel’s internal and external osmotic pressure differences are significant; therefore, the intermolecular binding force is small. As the water absorption increases, the molecular chains are stretched apart by water molecules, and the intermolecular binding force gradually increases. However, as the osmotic pressure gradually decreases, the water absorption rate slows down, which makes the expansion rate of the gel gradually slow down. After a certain period, the intermolecular binding forces and osmotic pressure within the gel reach equilibrium, and the volume of the gel is stable. From 3 to 7 days, the volume of the gel increases slowly. It is since the internal water absorption of the gel is less than the external water absorption per unit of time. Therefore, it takes longer for the internal and external water absorption of the gel to reach equilibrium, resulting in a slower rate of increase in the expansion rate.

Unfortunately, the expansion rate of the slurry with high-content bentonite and AC has decreased; however, it is enough to meet the requirements of grouting works. The increase in AC and bentonite content can improve the stability of the slurry, which makes the slurry more suitable for dynamic water sealing projects.

### 3.3. Impermeability

**Figure 15:**
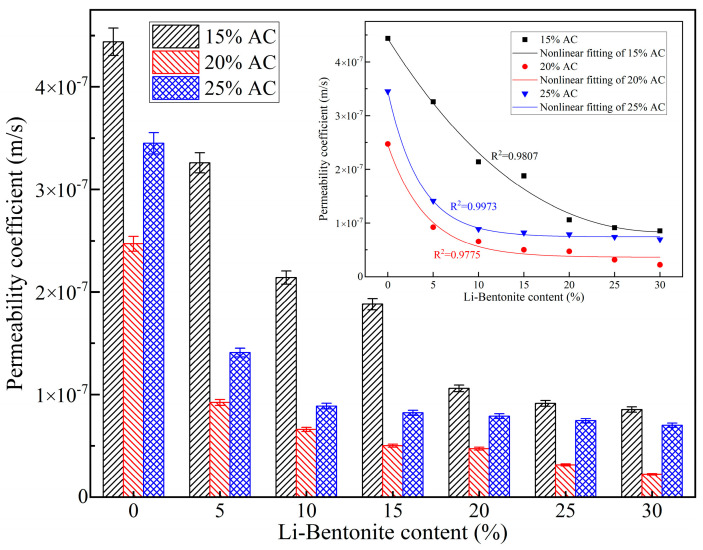
Impermeability of LiB-AC grout.

Anti-seepage performance is an important parameter of grouting materials, which can improve the sealing effect and durability of dynamic water grouting engineering. The impermeability of LiB-AC grout with different admixtures of Li-B and AC is shown in Figure 15.

It can be observed from Figure 15 that, when the AC content is the same, with the increase in Li-B content, the anti-seepage performance of LiB-AC grout gradually improves, with a minimum permeability coefficient of 2.23 × 10^−8^ m/s. In the gel process of AC slurry without Li-B addition, the linear polymer chain formed by polymerization will cross-link under the action of MBA, forming a dense spatial network of polymer structure. This three-dimensional network spatial structure can be linked between the Li-B particle pores, blocking the space’s flow intervals. With increased Li-B content, the seepage channel inside the gel becomes narrower. Therefore, the higher the bentonite content, the better the anti-seepage performance of the gel.

However, when the content of bentonite is constant, the permeability coefficient of the gel decreases first and then increases with the increase in AC content. When the AC content is 20%, the permeability coefficient of the gel is the smallest. It may be attributed to the spatial network structure formed by AC and bentonite particles. When the AC content is low, this spatial network structure becomes denser as the AC content increases. As the AC content increases, on the one hand, the increase in cation concentration in the slurry leads to a decrease in the thickness of the double layer on the surface of Li-B and a thinning of the firmly bound water layer adsorbed on its surface. Thus, the pores of bentonite particles increase, and the permeability coefficient increases accordingly. On the other hand, the cations on the polymer chain increase with the increase in AC content. Increased AC content leads to increased cations in the polymer chain. Cations could accelerate the dissociation of hydrophilic groups in the slurry, thereby increasing the number of anions. The high ion concentration in the slurry increases the electrostatic repulsion between them. As a result, the spatial network structure of the gel becomes relaxed, and the permeability coefficient increases.

Moreover, from the nonlinear fitting curves in Figure 15, it can be concluded that in the group with an AC content of 15%, the permeability coefficient of the gel decreases gently with the increase in bentonite content. In the group with an AC content of 20% and 25% and a bentonite content increasing from 0 to 15%, the permeability coefficient of the gel decreases sharply. After 15%, with the increase in bentonite content, the permeability coefficient of the gel tends to be stable. This indicates that when the AC content in the slurry is low, the permeability coefficient of the gel is affected by both bentonite and AC. However, when the AC content is high, the cross-linked network structure formed by AC is already tight enough, and a small amount of bentonite can be added to achieve the optimal anti-seepage effect.

### 3.4. Mechanical Strength

#### 3.4.1. Compressive Strength

**Figure 16:**
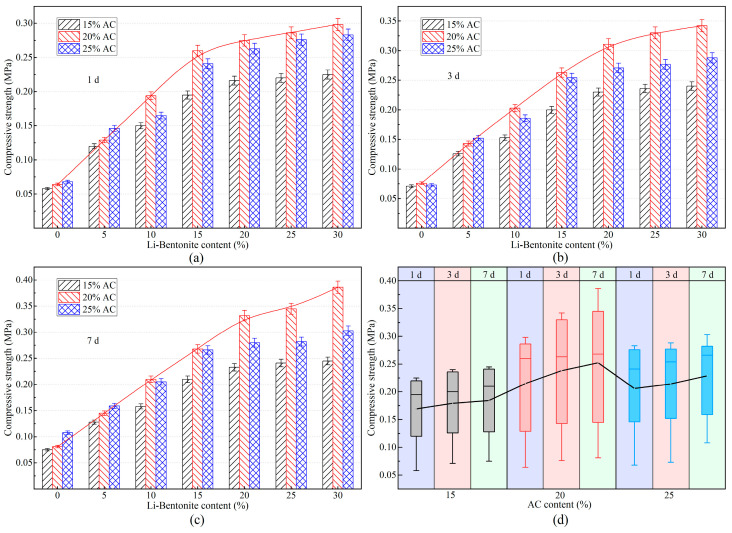
The compressive strength of LiB-AC grout: (**a**) curing time 1 day; (**b**) curing time 3 day; (**c**) curing time 7 day; (**d**) compressive strength of grout gel with different AC content under different curing time.

In the dynamic water grouting project, the LiB-AC grout should withstand a certain water pressure, which requires the gel to have high compressive strength. The compressive strength of LiB-AC grout with different admixtures of AC and Li-B at 1, 3, and 7 days is shown in Figure 16.

As shown in Figure 16a–c, the compressive strength of the LiB-AC improved as the content of bentonite increased, regardless of whether the curing time was 1 d, 3 d, or 7 d. It is the reason that the increase in Li-B content makes the spatial network structure of the slurry gel more compact. However, as the bentonite content increases, the growth rate of gel compressive strength changes from fast to slow. It may be due to the close saturation of the connection point between the acrylate crosslinker and bentonite in the gel.

However, when the content of bentonite is constant, the compressive strength of the gel increases first and then decreases with the increase in AC content, which is consistent with that of impermeability. It is suggested that there is an optimal density for polymer cross-linked networks, and an appropriate amount of AC content is beneficial for the structural stability of the cross-linked network. However, excessive cross-linking points reduce the strength of the spatial network formed in the slurry, making it prone to fracture.

Besides, it can be seen from Figure 16d that the compressive strength value of the LiB-AC gel curing for 7 d was higher than those for 1 and 3 d at the same content of AC and Li-B. The AC content of the 20% group showed the most significant increase in compressive strength with the change in curing time, with a maximum increase of 29.5%. This value is only 6.67% and 7.07% in the AC15% and AC25% groups.

#### 3.4.2. Tensile Strength

**Figure 17:**
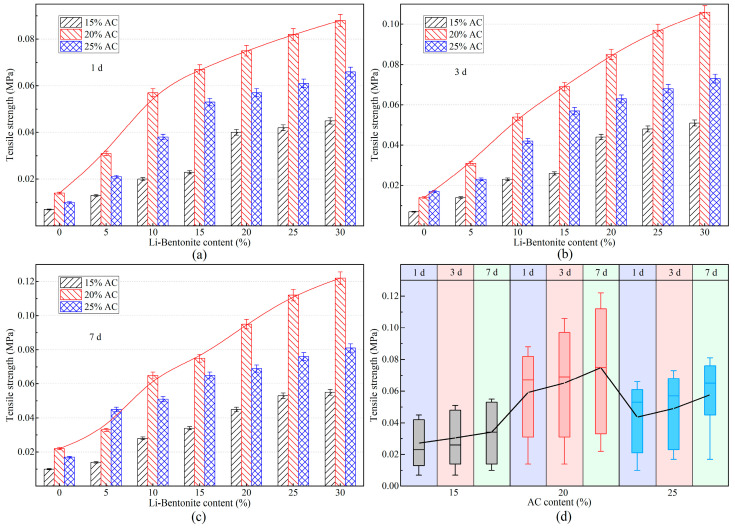
The tensile strength of LiB-AC grout: (**a**) curing time 1 day; (**b**) curing time 3 day; (**c**) curing time 7 day; (**d**) tensile strength of grout gel with different AC content under different curing time.

The grouting material gel should have a certain tensile strength to resist the shear stresses generated by structural misalignment and soil thawing subsidence. The tensile strength of LiB-AC grout with different admixtures of AC and Li-B at 1, 3, and 7 days is shown in Figure 17.

It can be concluded from Figure 17a–c that the tensile strength of the LiB-AC improved as the content of bentonite increased, regardless of whether the curing time was 1, 3, or 7 d. It is the reason that the increase in Li-B content makes the spatial network structure of the slurry gel more compact. And the maximum tensile strength of the gel reached 0.088 MPa. However, with the increase in bentonite content, the growth rate of gel tensile strength changes from fast to slow. It may be due to the close saturation of the connection point between the acrylate crosslinker and bentonite in the gel.

Nevertheless, when the content of bentonite is constant, the tensile strength of the gel increases first and then decreases with the increase in AC content. This phenomenon is consistent with those of impermeability and compressive strength. It can be inferred that there is an optimal density for polymer cross-linked networks, and an appropriate amount of AC content is beneficial for the structural stability of the cross-linked network. However, excessive cross-linking points reduce the strength of the spatial network formed in the slurry, making it prone to fracture.

Moreover, as shown in Figure 17d, the tensile strength value of the LiB-AC gel curing for 7 d was higher than those for 1 and 3 d at the same content of AC and Li-B. The AC content of the 20% group showed the greatest increase in tensile strength with the change in curing time, with a maximum increase of 29.5%. Therefore, in conjunction with the conclusions obtained from the compressive strength test, an AC content of 20% in LiB-AC grout is most beneficial for the mechanical strength of the slurry.

### 3.5. Microstructural Characteristics

#### 3.5.1. XRD Analyses

**Figure 18:**
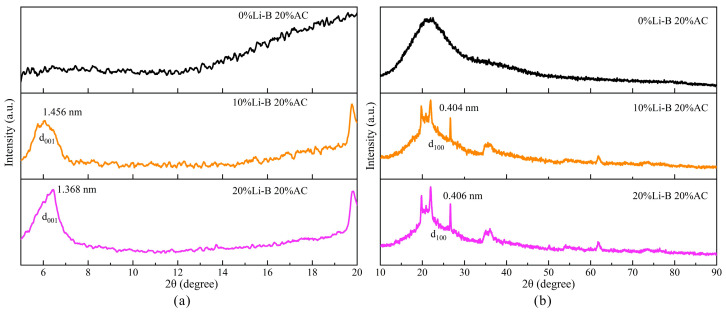
XRD spectra of LiB-AC grout: (**a**) scanning range is 5–20°; (**b**) scanning range is 10–80°.

The distance between lamellae of Li-B is called the lamellar spacing, which can be calculated based on the Bragg equation. If there is no intercalation phenomenon between Li-B and AC, the lamellar spacing of Li-B will not change, and it shows a change in peak position angle on the XRD spectrum. However, when the Li-B lamellae are peeled off, the interlayer is completely stretched, and there will be no characteristic peaks. The scanning range of this XRD experiment is 5–20° and 10–80°, as shown in Figure 18.

Figure 18a shows that after intercalation with Li-B, AC exhibits a new characteristic peak at around 6°. It can be inferred that Li-B and AC have completed intercalation polymerization, and Li-B is evenly distributed in AC. When the content of Li-B increases from 10% to 20%, the d_001_ peak of Li-B shifts from 6.06569° to 6.45508° with a large angle, and the lamellar spacing decreases from 1.456 to 1.368 nm. As Li-B content increases, the spacing between the Li-B lamellae decreases instead. It indicates that the degree of exfoliation of Li-B is weakened, and the lamellae are more spatially compacted.

The typical diffraction profile of the AC polymer is shown in Figure 18, with a diffuse bun peak to the right at 20°. After adding Li-B, the d_100_ peaks of 10% LiB-AC and 20% LiB-AC changed. The characteristic peak shape of Li-B has appeared. It may be due to the interaction between the acrylate polymer and the surface charge of bentonite, which increases the degree of detachment of the bentonite crystal lamella. Meanwhile, the d_100_ peak shape of 20% LiB-AC is sharper and wider than that of 10% LiB-AC, and the position of the peak remains the same. It indicates that the polymerization modification did not change the lamellar structure of the bentonite, and the degree of peeling of the bentonite increased.

#### 3.5.2. IR Analyses

**Figure 19:**
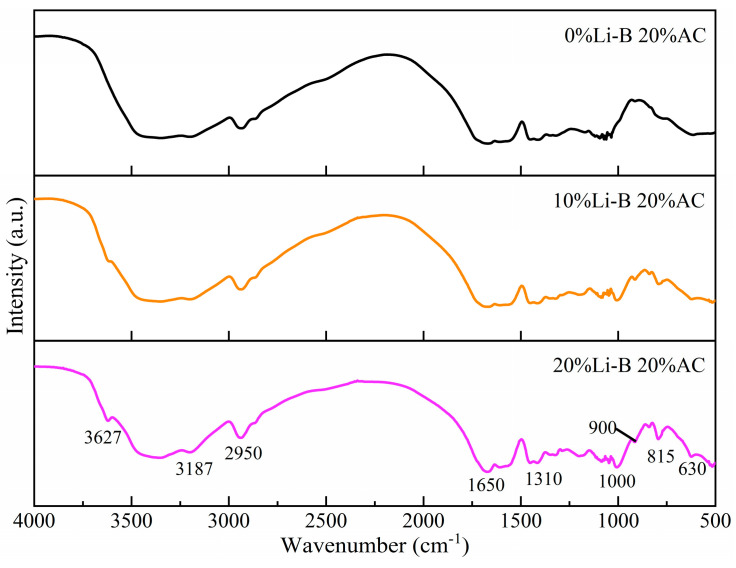
IR spectra of LiB-AC grout.

The chemical bond characteristics of LiB-AC grout with different dosages of Li-B are analyzed by infrared spectra, as shown in Figure 19.

It can be observed from Figure 19 that the basic framework of the polymer is still the network structure of AC, and no new characteristic peaks appeared. It is suggested that the addition of Li-B has almost no effect on the spatial structure of AC. However, after adding Li-B, different characteristic peaks appeared in the slurry with 10% and 20% bentonite content. The new characteristic peaks are as follows: The peak at 3627 cm^−1^ of Li-B is the stretching vibration of O-H on AL-O-H; the peak at 3187 cm^−1^ is the stretching vibration of C-H; the peak at 2950 cm^−1^ is the asymmetric vibration of -CH_3_; the peak at 1650 cm^−1^ is the superposition of the bending vibration of water molecule H-O-H and the absorption of C=C. The vibration peak at 1310 cm^−1^ is poly-hydrocarbon C-C, and 1000 cm^−1^ is the anti-symmetric stretching vibration peak of Si-O-Si. The peak at 900 cm^−1^ is the vibrational contraction of -OH in Li-B, and 815 cm^−1^ is the absorption peak of Si-O-Si. The peak at 630 cm^−1^ is the enhanced absorption caused by the sulfate generated by ammonium persulfate in the reaction.

#### 3.5.3. SEM Analyses

**Figure 20:**
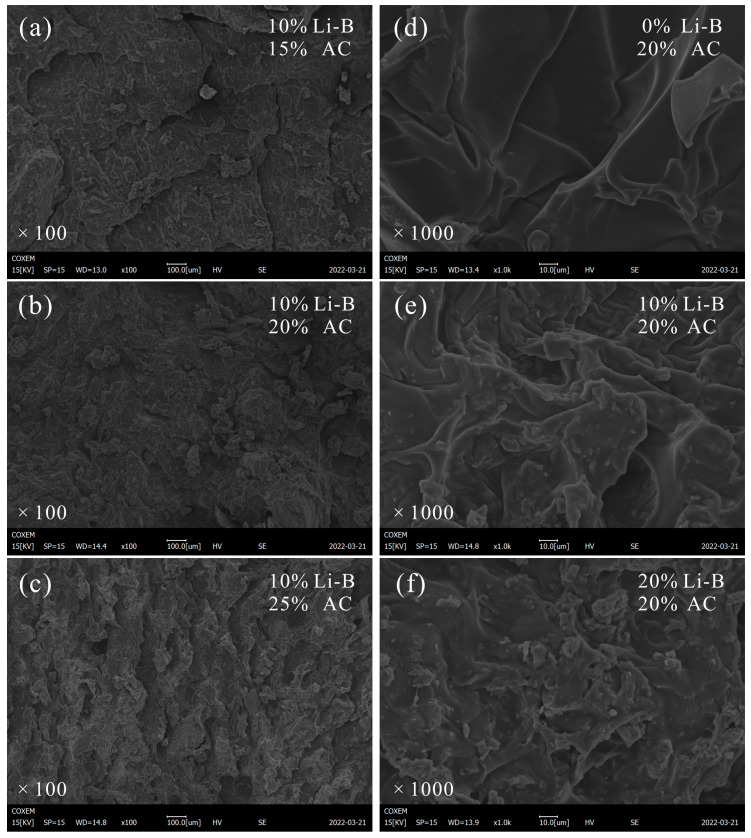
SEM of LiB-AC grout: (**a**) zoom in 100 times, 10% Li-B and 15% AC; (**b**) zoom in 100 times, 10% Li-B and 20% AC; (**c**) zoom in 100 times, 10% Li-B and 25% AC; (**d**) zoom in 1000 times, 0% Li-B and 20% AC; (**e**) zoom in 1000 times, 10% Li-B and 20% AC; (**f**) zoom in 1000 times, 20% Li-B and 20% AC.

In order to further examine the anti-seepage performance and change the law of mechanical strength, SEM analysis was employed to characterize and compare the microstructure and composition of LiB-AC gel mixed with different dosages of Li-B or AC, as shown in Figure 20.

Comparing Figure 20a–c, Figure 20b has the most complete gel structure and the smoothest surface. Large lamellar structures can also be seen in Figure 20a. With the increase in AC content to 25%, the surface of the gel becomes very rough, and the pores become larger. As mentioned in Section 3.3 and Section 3.4, this confirms why the impermeability properties and the mechanical strength of the gels show a pattern of increasing and then decreasing with increasing AC content.

It can be seen from Figure 20d–f that with the increase in bentonite content, the surface of AC gel gradually becomes rough. And AC wraps Li-B tightly, indicating that bentonite is compatible with AC. The LiB-AC grout without bentonite was relatively dense, and the gel of the LiB-AC grout containing bentonite was loose, which may be bentonite’s dispersion and packing effect on the AC polymer. Owing to the interlocking network structure formed by the combination of Li-B and AC, the slurry has good resistance to dynamic water washout, impermeability, and mechanical strength.

### 3.6. Modification Mechanism of Lithium Bentonite on Acrylate Slurry

**Figure 21:**
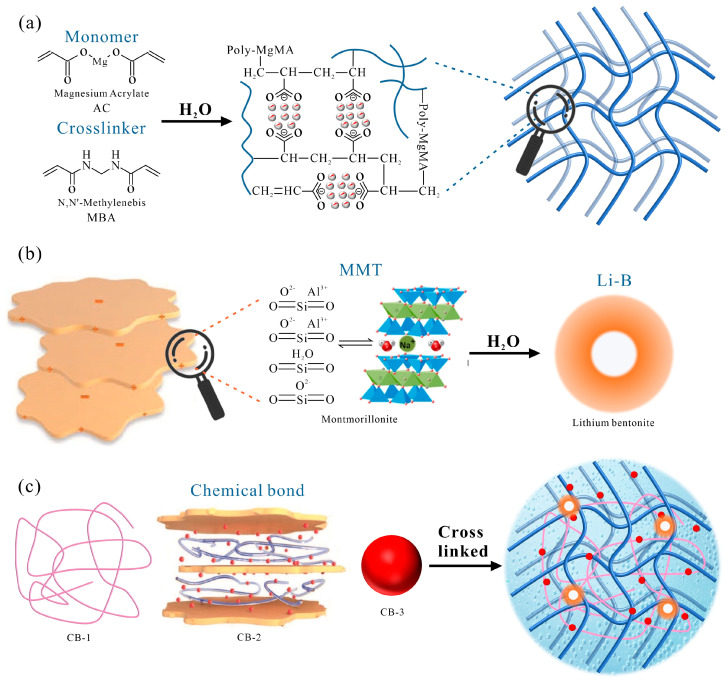
Modification mechanism of Li-B on AC slurry: (**a**) magnesium acrylate forms a cross-linked network; (**b**) hydration of bentonite; (**c**) graft reaction between cross-linked network and bentonite.

Based on the abovementioned performance test results and microstructure analysis of the LiB-AC grout, a conceptual structural model was proposed to interpret the modification mechanism of Li-B on AC slurry. This structural model is illustrated in Figure 21, and the synthesis of this structure can be divided into three steps: the formation of an acrylate network structure, the water absorption and dispersion of Li-B, and the combination of the two materials mentioned above.

First, by adding the initiator (ammonium persulfate) to the acrylate solution, the acrylic monomer breaks the double bond and polymerizes into a linear chain under the action of APS. Linear magnesium polyacrylate generates cross points and forms cross-branched chains under the action of MBA [38,39]. These branches are interconnected to form a three-dimensional network structure, as shown in Figure 21a.

Second, the exchangeable cations and water molecules enter the interlayer after mixing bentonite with water [40]. At the same time, due to the cation hydration between the bentonite lamella, the adsorbed water molecules form a hydration film, which increases the lamellar spacing and causes expansion. As montmorillonite particles are negatively charged and repel each other, bentonite particles are suspended in water, as shown in Figure 21b.

Besides, after the combination of AC and bentonite, a staggered spatial structure is formed with the AC network structure as the skeleton, and bentonite is grafted onto it, as shown in Figure 21c. This structure is mainly combined in the following three ways: (1) Acrylate is wrapped with bentonite particles, forming a staggered spatial structure (CB-1). (2) Acrylate chains are intercalated into the bentonite lamella and connected through van der Waals forces and hydrogen bonds (CB-2). (3) Acrylate undergoes cation exchange with bentonite and is connected to the surface of bentonite with hydrogen bonds (CB-3).

Furthermore, Li-B has a smaller radius of Li ions and a tighter binding with the acrylate network than common calcium and sodium bentonite [41]. Therefore, this staggered spatial network structure makes the performance of the slurry more stable, making it suitable for dynamic water grouting environments.

## 4. Engineering Application

The shield tunnel structure of a metro is located in a water-rich sandy pebble stratum. Due to the rising groundwater levels and the misalignment of the shield tunnel segment, water leakage points have emerged, seriously affecting the regular operation of the metro.

After on-site investigation, the tunnel to be treated has a buried depth of 18 m, and the groundwater level is about 10 m higher than the tunnel arch. Ensuring effective slurry retention under high water pressure is an urgent problem to be solved. Cement-water glass (C-S) and LiB-AC grout were both adopted for comparison in the pre-grouting application. The first grout was C-S, slurry A was the cement slurry, with a water/cement ratio of C-S grout of 1:1, and slurry B was 35°Bé water glass. The volume ratio of components A and B was 1:1. The second grout is LiB-AC; slurry A was 30% bentonite and 20% AC, and slurry B was a solution of APS with a mass fraction of 1%. The third slurry does not add bentonite, and other parameters are consistent with the second.

**Figure 22:**
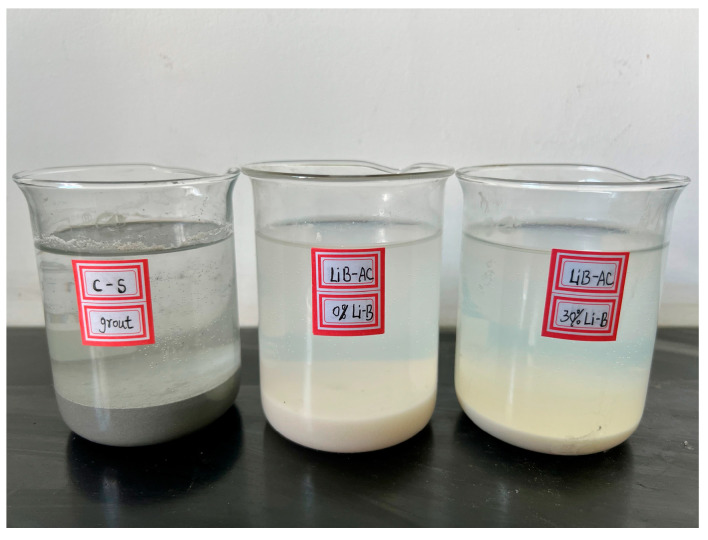
The grouting off target characteristics of C-S and LiB-AC grout.

The retention rate of C-S and LiB-AC grout is determined by collecting water at the leakage point using a beaker, as shown in Figure 22. It is outstanding that the slurry retention rate of LiB-AC grout was higher than that of C-S.

**Figure 23:**
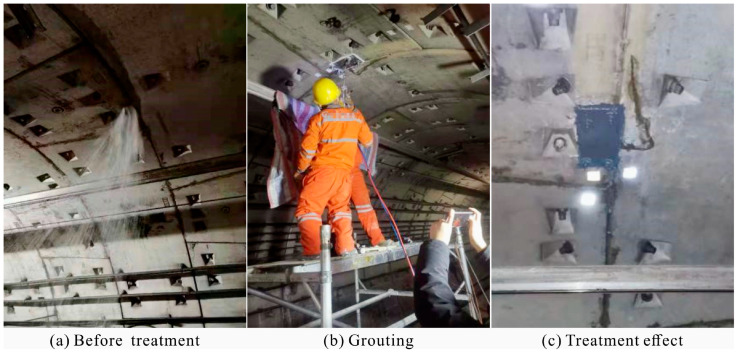
Sealing effect of LiB-AC grout.

And then, LiB-AC grout is used as the primary sealing material, and a back cover grouting machine is used for grouting, with a grouting pressure of 1 MPa. The sealing effect is shown in Figure 23. The engineering application indicated that LiB-AC grout could seal the dynamic water in high-velocity aquifers.

## 5. Conclusions

In this study, we have developed a novel and highly efficient dynamic water grouting material referred to as LiB-AC grout. The grouting materials’ workability, mechanical properties, microstructures, and the mechanism for their improved washout resistance were studied. Furthermore, this innovative material was applied in a shield tunnel segment grouting project, which successfully addressed the dynamic water leakage of the tunnel. The following conclusions can be obtained:

(1) LiB-AC grout has the advantages of adjustable setting time (10.5 to 395.6 s), minimal bleeding (0.1%), high viscosity (65 mPa·s) and expansibility (350%), robust dynamic water anti-dispersion performance (24 times that of pure AC slurry), high mechanical strength (compressive strength is 0.386 MPa, tensile strength is 0.088 MPa), impermeability (2.23 × 10^−8^ m/s), and non-toxic attributes.
(2) The lithium bentonite was beneficial to the setting time, bleeding, viscosity, slurry retention rate, impermeability, and mechanical strength of the acrylate grout. However, it diminished the expansibility of the acrylate grout. At the optimal acrylate content (20%), the mechanical strength and impermeability of the LiB-AC grout were the highest.
(3) The better performance on washout resistance of LiB-AC grout is from the modification of acrylate by bentonite, which leads to the formation of an interwoven spatial network structure of the LiB-AC grouting material. Besides, the smaller lithium-ion radius of lithium bentonite contributes to the enhanced stability of the network structure.
(4) The LiB-AC grout succeeded in leakage prevention in a shield metro tunnel segment grouting project in a water-rich sandy pebble stratum. On-site application of the LiB-AC grout showed that it had a better anti-washout performance compared with cement-water glass and pure AC grout.

## Figures and Tables

**Table 1:** The chemical composites of Li-B (in mass %).

SiO_2_	Al_2_O_3_	MgO	Na_2_O	CaO	K_2_O	Fe_2_O	TiO_2_	Li_2_O	Loss
65.89	13.60	3.79	0.31	1.94	0.58	1.80	0.23	0.74	10.79

## Data Availability

The data presented in this study are available on request from the corresponding author.
